# Socioeconomic determinants of early childhood development: evidence from Pakistan

**DOI:** 10.1186/s41043-024-00569-5

**Published:** 2024-05-20

**Authors:** Shahla Akram, Feroz Zahid, Zahid Pervaiz

**Affiliations:** 1 Department of Economics, National College of Business Administration and Economics, Lahore, Pakistan; 2 Al-Aleem Medical College, Lahore, Pakistan

**Keywords:** Early childhood development, Child malnutrition, Socioeconomic status, Parental education, Child disabilities

## Abstract

This study investigates the socioeconomic determinants of early childhood development (ECD) in Pakistan by utilizing the data of sixth wave of the Multiple Indicator Cluster Survey (MICS) conducted in the four provinces of the country. The findings of the study reveal that mother’s education, father’s education, economic status of the household as measured by household’s wealth index quintile, region of residence (province), child’s gender, disability, nutrition and the practices used by the adult members of the household to discipline child are important determinants of ECD. The study highlights the crucial role of family background and importance of addressing the issue of malnutrition to foster child development.

## Introduction

Early childhood development (ECD) encompasses child’s social, cognitive and physical development during early childhood period. ECD is considered important as it lays foundations for child’s overall growth as well as success in the subsequent years of life. Early years of life are crucial for the brain development of the child [21] and investment in early years of childhood have the highest economic returns in low income as well as high income countries [34]. Because of the importance of ECD, it is a part of United Nations sustainable development goal (SDG) 4 which aims to “ensure inclusive and equitable quality education and promote lifelong learning opportunities for all”. Specifically, the indicator 4.2.1 is devised to deal with ECD. Approximately 43% of children under the age of five worldwide face developmental risks due to poverty, hunger, and limited access to essential services whereas early childhood malnutrition affects 200 million children worldwide.^1^ This can significantly impact child's well-being and future prospects. To ensure optimal child development, timely interventions are essential. However, many developing countries lack substantial policy interventions and comprehensive ECD initiatives.

ECD can be affected by various biological as well as socioeconomic factors. Numerous studies have established a connection between socioeconomic factors and developmental outcomes of newborns, infants, and children during early childhood [5, 20, 30–32]. Child raised in low-income households are more prone to cognitive and behavioral challenges [10] whereas higher levels of parental education are associated with positive developmental outcomes including socioemotional and cognitive development [15, 29, 35]. Parental occupation can shape child’s developmental paths, as parents with more rewarding professions are more likely to adopt enriching parenting practices [2, 14]. While it is widely recognized that lower socioeconomic status of parents negatively affects behavioral, cognitive, and linguistic domains of child development, the role of parental education can also be of significant importance as the intersection of parental education with other socioeconomic factors can work to shape such development [3]. Furthermore, child's gender may also be important determinant of ECD due to sociocultural norms associated with gender [6, 11]. The environment in which a children grow up is important to shape ECD, as economic, cultural, and social differences can affect child’s well-being and social growth [19, 36]. Practices adopted by adults to discipline the child in the household can significantly impact a child's mental health, cognitive development, and behaviour [8, 11, 12, 28]. Another crucial factor with substantial developmental consequences is child nutrition. Malnutrition during childhood can lead to stunted growth, cognitive impairment, and increased susceptibility to infections [7, 8, 22]. Extensive research has explored the negative impacts of malnutrition on cognitive development, with studies suggesting the potential benefits of nutrient diet in improving cognitive, linguistic, and motor skills [4, 30].

This study aims to investigate the socioeconomic determinants of ECD by utilizing the data of sixth wave of MICS conducted in four provinces of Pakistan. The country’s performance in terms of different development indicators and in achieving sustainable development goals has not been satisfactory. Exploring factors affecting ECD can provide important insights which can be helpful for policy makers to adopt suitable policy interventions.

## Methodology

ECD is influenced by different factors, including parental and household’s socioeconomic status as well as child’s individual characteristics. The Multiple Indicator Cluster Survey (MICS), a comprehensive household survey conducted in collaboration between UNICEF and the Bureau of Statistics of Pakistan as part of the Global MICS wave 6 Programme has been used to investigate these determinants of ECD.^2^ The survey was conducted in four provinces of Pakistan during the period of 2017–2020. The sample of survey data collected for children under the age of five years, consisted of 25,638 in Balochistan, 24,345 in KPK, 18,312 in Sindh, and 42,408 in Punjab. However, in our analysis, we utilized the data of children aged above three and less than five years (36–59 months), the sample of which consisted of total 39,168 children in Pakistan. After removing the missing observations in our data, we had comprehensive information of 36,947 children which was used in our empirical analysis. Our dependent variable is early childhood development index (ECDI) whereas independent variables include mother’s education, father’s education, wealth index quintile used as a proxy of household’s economic status, area of residence (rural/urban), region of residence (province), child’s gender, child’s disability and child discipline (methods and practices used by the adults of the household to discipline child). The methodology of United Nations Children’s Fund (UNICEF) has been followed to develop ECDI which is also termed as MICS ECDI [18]. The ECDI is constructed by using ten items/indicators designed specifically for children aged 36–59 months [17]. These indicators are related with four broader domains which include literacy-numeracy, social-emotional, physical and learning. Children are considered to have literacy-numeracy skills if they demonstrate a minimum of two out of three learning behaviours, such as recognizing and naming a minimum of 10 letters of the alphabet, reading at least four common words, and recognizing numbers from 1 to 10 along with their corresponding symbols. Social-emotional development is assessed by behaviours such as harmonious interactions with peers, refraining from aggressive behaviours like kicking or biting, and maintaining focus. Physical development is judged by the child's ability to grasp a small object using two fingers and/or through mother/primary caregiver’s confirmation of child’s ability to play without feeling sick. Learning development is established by the child's ability to follow simple instructions for task execution and their capacity for autonomous task completion. Child is considered developmentally on track if he/she can demonstrate his/her ability in at least three out of four domains. The ECDI is a binary variable which is code as 1 if child is developmentally on track and 0 otherwise. Mother’s education is coded as 0 if she has lesser than secondary education and coded as 1 if has secondary or above education. Father’s education is also a binary variable and takes the value 1 in case of at least secondary education otherwise 0. Wealth index quintile used as a proxy of household’s economic status is our third independent variable. MICS constructs wealth scores for households based on factors including households’ access to essential services, possession of durable goods, and household characteristics. On the basis of calculated wealth scores, households are placed in five quintiles. The poorest households are placed in the bottom or first quintile whereas the richest households are placed in the top or fifth quintile. The variable has been used as multiple category dummy variable with first wealth quintile used as reference category. The fourth independent variable is area of residence coded as 0 in case of rural and 1 in case of urban households. Region of residence is a multiple category dummy variable categorized into four provinces named as Balochistan, Punjab, Sindh and Khyber Pakhtunkhwa (KPK). Child gender is a dummy variable coded as 0 in case of female and 1 in case of male child. The seventh independent variable is child disability coded as 0 in case of any functional disability such as seeing or hearing and coded as 1 if there is no such disability. Child discipline is the next variable which has been measured through the methods and practices adopted to discipline child within household. The variable is coded as 0 in case of some violent practices are used and 1 if no such practices are used. The last and ninth independent variable is malnutrition which has been used as dummy variable coded as 0 if child experiences stunted growth and 1 otherwise.

Although malnutrition manifests in various ways, including wasting, stunting, underweight, overweight or obesity yet Pakistan exhibits alarming severity in stunting and have lesser issues of overweight, obesity, and other forms of malnutrition.^3^ Therefore, we have operationalized the variable of malnutrition through stunting which has been measured using height-for-age z-scores (≤ -2 standard deviations) by following WHO (World Health Organization) guidelines.^4^ The dependent variable of our study is binary variable. In such situation, the use of logistic regression is appropriate instead of ordinary least square (OLS). Therefore, we have used logistic regression for our empirical analysis. The empirical analysis has been performed by using stata 17.

## Results

Table 1 below provides the cross-tabulation of our data.

**Table 1:** Cross-tabulation of data

	Early childhood development (%)		
	No	Yes	Total
*Mother’s education*			
Less than secondary	53.77	31.02	84.79
At least secondary	7.96	7.25	15.21
*Father’s education*			
Less than secondary	41.95	24.09	66.04
At least secondary	19.77	14.18	33.96
*Wealth index quintile*			
First	18.73	10.02	28.75
Second	14.10	8.60	22.71
Third	11.84	7.44	19.28
Fourth	9.92	6.48	16.40
Fifth	7.13	5.73	12.86
*Area*			
Rural	46.93	27.66	74.59
Urban	14.80	10.61	25.41
*Province*			
Balochistan	19.58	6.75	26.34
Punjab	18.23	17.06	35.29
Sindh	10.83	7.20	18.03
KPK	13.08	7.26	20.34
*Child gender*			
Female	30.17	17.95	48.11
Male	31.56	20.32	51.89
*Child disability*			
Yes	3.55	0.66	4.21
No	58.18	37.61	95.79
*Child discipline*			
Violent	23.33	8.00	31.32
Nonviolent	38.40	30.28	68.68
*Malnutrition*			
Stunted	28.42	15.24	43.67
Not stunted	33.31	23.03	56.33

The Table 1 provides information about different variables used in our analysis. The statistics provided in Table 1 show that majority of mothers in our sample (84.79%) have less than secondary school education. In case of father’s education, the situation is slightly better. About 38% of the children are developmentally on track whereas 43.67% are suffering from stunted growth. Majority of the respondents belong to rural area and are from Punjab province which is the largest province with respect to population. The female children are 48.11% of the sample whereas 51.89% children are male. 4.21% children have some functional disability. The empirical results of our logistic regression have been reported in the Table 2.

**Table 2:** Socioeconomic determinants of early childhood development (dependent variable: ECD)

Variables	Odd ratios	Marginal effect
*Mother's education*		
Less than secondary	Reference	
At least secondary	1.170***	1.036***
	(0.0423)	(0.00849)
*Father's education*		
Less than secondary	Reference	
At least secondary	1.055**	1.012**
	(0.0280)	(0.00597)
*Wealth index quintile*		
First	Reference	
Second	1.081**	1.017**
	(0.0341)	(0.00708)
Third	1.059*	1.013*
	(0.0362)	(0.00763)
Fourth	1.048	1.010
	(0.0407)	(0.00865)
Fifth	1.208***	1.043***
	(0.0563)	(0.0109)
*Area*		
Rural	Reference	
Urban	1.018	1.004
	(0.0305)	(0.00667)
*Province*		
Balochistan	Reference	
Punjab	2.134***	1.184***
	(0.0651)	(0.00782)
Sindh	1.691***	1.121***
	(0.0603)	(0.00870)
KPK	1.363***	1.067***
	(0.0470)	(0.00776)
*Child gender*		
Female	Reference	
Male	1.085***	1.018***
	(0.0240)	(0.00498)
*Child disability*		
Yes	Reference	
No	2.674***	1.209***
	(0.193)	(0.0137)
*Child discipline*		
Violent	Reference	
Nonviolent	2.006***	1.164***
	(0.0512)	(0.00617)
*Malnutrition*		
Stunted	Reference	
Not stunted	1.107***	1.023***
	(0.0255)	(0.00522)
Constant	0.0762***	
	(0.00588)	

SE in parentheses and ***p < 0.01, **p < 0.05, *p < 0.1

The results show that impact of mother’s education on early childhood development is significant, with higher levels of maternal education are associated with increased childhood development. This highlights the vital role of mother's education for child's growth. Similarly, there is a positive relationship between father's education and early childhood development. However, it is evident from reported odd-ratios that the effects of mother’s education are more pronounced than the effects of father’s education on ECD. Economic status of the households as measured by the wealth index quintile, is another crucial factor for ECD. The likelihood of early childhood development increases with improvements in the economic status of the households, indicating better developmental outcomes for children from wealthier households. Area of residence (rural/urban) has not been found to play a significant role in explaining the differences of ECD. However, region of residence (province) has been found to be crucial. Children from Punjab have the highest likelihood of ECD followed by the children residing in Sindh, KPK and Balochistan, highlighting the existence of regional inequality. Gender also plays a role, with male children having slightly higher probabilities of ECD. Child disabilities significantly impact developmental trajectories, as children with disabilities are less likely to have ECD as compared to their peers who do not have any kind of disabilities. The household environment also matters significantly, as children who do not have to face any violence in their households have higher likelihood of ECD compared to those who have to face violence in households. Moreover, malnutrition has a strong negative relationship with ECD. Stunted children have lower likelihood of proficiency in child developmental compared to non-stunted peers, emphasizing the critical role of proper nutrition in fostering physical growth, learning, and cognitive development. These findings highlight the crucial role of various socioeconomic determinants, including parental education, wealth status, child disability, region of residence and household environment, in shaping ECD. The adverse link between stunting and ECD underscores the paramount importance of addressing nutritional aspects for child’s physical growth and cognitive advancement.

## Discussion

This study provides valuable insights into the different socioeconomic determinants of ECD. A significant finding is the strong positive relationship between parental education and ECD, aligning with prior research highlighting the pivotal role of parental education in fostering child's physical, cognitive and socioemotional development [16, 25]. Parents with higher education levels are more likely to create stimulating environments and employ effective parenting techniques, fostering more successful child development [13]. Additionally, the study emphasizes the significant role of socioeconomic factors in shaping child’s development [37]. While the role of parental education is vital, it is important to note that the effects of mother’s education on ECD are greater than father’s education which further reinforce the importance of educated mothers for child’s development. While existing literature highlights the role of mother’s education for several developmental outcomes of child [2, 1, 23, 24] it may matter a lot for ECD because child care in their early years is considered the prime responsibility of mother particularly in Pakistani context. Furthermore, the study highlights the significance of economic status of the households and residence in relatively developed regions of the country for ECD. Economic status of the households and living in relatively developed regions positively correlate with better developmental outcomes, underscoring the importance of resource access, quality healthcare, and educational opportunities [9, 26]. The study also reveals negative associations of child disability and malnutrition with developmental outcomes, aligning with previous research emphasizing the detrimental impact of malnutrition and disabilities on cognitive and physical development [33]. Addressing these challenges through targeted interventions can be helpful for improving child’s developmental outcomes as previous research highlight the role of social protection programs in mitigating poverty-related adversities on child development [27].

## Conclusion

The study sheds light on the role of different socioeconomic factors for early childhood development. The findings underscore the importance of parental education and household’s economic status as important determinants of early childhood development. It further highlights the significant role of violence free household environment and proper nutrition for Child's cognitive, physical, and socioemotional development. Moreover, gender of child, disability and region of residence have also been found important for ECD. We particularly suggest some policy options and intervention strategies. There is a need to design policies to enhance the access to education facilities especially for girls. It would not only bridge the gaps of developmental outcomes across genders but also be beneficial for future generation’s development due to significant role of mother’s education for ECD. Policies to reduce income inequality as well as regional inequality can also be important. Social support initiatives are also required to mitigate poverty related adverse effects for child development.

## Data Availability

We have used the secondary data of MICS wave 6 available at https://mics.unicef.org/surveys.

## Footnotes

1 https://www.unicef.org/pakistan/early-childhood-development-ecd-0

2 https://mics.unicef.org/surveys

3 https://blogs.worldbank.org/endpovertyinsouthasia/addressing-pakistans-high-level-stunting-now

4 https://www.who.int/health-topics/malnutrition#tab=tab_1
